# Efficacy and Toxicity of CDK4/6 Inhibitors in Early and Metastatic HR+/HER2− Breast Cancer: An Updated Meta-Analysis of Phase III Trials

**DOI:** 10.3390/cancers18111714

**Published:** 2026-05-24

**Authors:** Ravneet K. Dhanoa, Sumin Thapa, Pragnan Kancharla, Shweta Kurian

**Affiliations:** 1 Department of Internal Medicine, MedStar Union Memorial Hospital, Baltimore, MD 21218, USA; 2 Department of Internal Medicine, MedStar Franklin Square Medical Center, Baltimore, MD 21237, USA; drsuminthapa@gmail.com; 3 Department of Hematology/Oncology, MedStar Franklin Square Medical Center, Baltimore, MD 21237, USA; pragnan.kancharla@medstar.net (P.K.); shweta.kurian@medstar.net (S.K.)

**Keywords:** hormone receptor-positive breast cancer, HER2-negative breast cancer, CDK4/6 inhibitors, early breast cancer, metastatic breast cancer, phase III trials

## Abstract

**Background/Objectives**: CDK4/6i and ET improve outcomes in HR-positive, HER2-negative breast cancer, but their benefits differ between early-stage (EBC) and metastatic disease (MBC). We aimed to compare the efficacy and toxicity of CDK4/6i plus ET versus ET alone across these disease settings. **Methods**: We conducted a meta-analysis of phase III randomized trials identified through MEDLINE, EMBASE, and Web of Science up to 1 October 2025. Twenty-two trials (18 MBC, n = 6364; 4 EBC, n = 17,741) met the inclusion criteria. Two reviewers extracted data and assessed risk of bias. The study followed PRISMA 2020 guidelines (PROSPERO: CRD420251132302). Outcomes included overall survival (OS), progression-free survival (PFS), invasive disease-free survival (iDFS), distant relapse-free survival (DRFS), response rates, treatment discontinuation (TDR), dose reductions (DRR), and grade 3–4 adverse events. **Results**: In MBC, CDK4/6i improved OS (HR: 0.78, 95% CI: 0.72–0.85) and PFS (HR: 0.53, 95% CI: 0.50–0.56) and increased objective response, clinical benefit, and disease control rates along with TDR, DRR, and grade 3–4 adverse events. In EBC, iDFS improved, whereas OS (HR: 0.92, 95% CI: 0.75–1.12; *p* = 0.40) and DRFS did not. TDR, DRR, and grade 3–4 adverse events were increased. **Conclusions**: CDK4/6i confer significant OS and PFS benefits in MBC, supporting first-line use. In EBC, no OS benefit was observed, and toxicity was increased, underscoring the need for individualized risk–benefit assessment. Longer follow-up is warranted to clarify survival outcomes.

## Simple Summary

Cyclin-dependent kinase 4/6 (CDK4/6) inhibitors have become a cornerstone for the treatment of hormone receptor-positive, HER2-negative breast cancer, yet their role differs between early-stage and metastatic disease. In this meta-analysis of 22 phase III trials, we found that in early-stage breast cancer, CDK4/6 inhibitors improved invasive disease-free survival but did not demonstrate a clear overall survival benefit and were associated with increased hematologic and non-hematologic toxicities, leading to higher treatment discontinuation and dose reductions. These findings highlight the importance of individualized risk–benefit assessment and the need for longer follow-up to determine survival impact in early-stage disease. In contrast, in metastatic disease, CDK4/6 inhibitors significantly improved overall survival, progression-free survival, and response rates, supporting their established role as standard first-line therapy. These results reinforce stage-specific treatment considerations in clinical practice.

## 1. Introduction

Breast cancer is the most common cancer among women worldwide [1]. It comprises molecular subtypes defined by hormone-receptor and human epidermal growth factor receptor 2 (HER2) status, of which hormone receptor-positive (HR+) and HER2-negative (HER2−) disease is the most prevalent [1,2,3]. HR+/HER2− breast cancer is biologically and clinically heterogeneous, encompassing both early-stage breast cancer (EBC) and metastatic breast cancer (MBC) [4]. EBC is potentially curable and managed with curative-intent multimodality therapy, including surgery, radiation therapy, and adjuvant endocrine therapy (ET) with or without chemotherapy [5]. MBC is incurable, with treatment focused on prolonging survival through sequential systemic therapies [1].

In the metastatic setting, ET remains the foundation of treatment [2]; however, despite initial clinical benefit, most patients ultimately develop acquired resistance, resulting in disease progression [1,6,7]. Aberrant activation of cyclin-dependent kinase 4 and 6 (CDK4/6)–retinoblastoma (Rb) signaling axis—through cyclin D overexpression, CDK4/6 amplification, or loss of endogenous CDK inhibitors—drives unrestrained G1-to-S phase cell-cycle transition, leading to tumor proliferation and resistance to ET [6,7,8].

CDK4/6 inhibitors target this dysregulation, restoring cell-cycle control and overcoming endocrine resistance [6,7,8]. By preventing Rb phosphorylation, these agents induce sustained G1 arrest and exert anti-tumor effects through modulation of tumor immunity and the tumor microenvironment [7,8,9]. Pivotal phase III trials demonstrated substantial improvements in progression-free survival (PFS), with pooled and long-term analyses subsequently confirming overall survival (OS) benefits, establishing CDK4/6 inhibitor-based combinations as standard first-line therapy in MBC [10,11,12]. Three oral CDK4/6 inhibitors—palbociclib, ribociclib, and abemaciclib—are approved in combination with ET for advanced HR+/HER2− breast cancer [8,9,11,12]. In contrast, evidence for CDK4/6 inhibitors in EBC has been heterogeneous. Adjuvant abemaciclib (monarchE) and ribociclib (NATALEE) significantly improved invasive disease-free survival (iDFS) in selected patients with high-risk or stage II–III HR+/HER2− breast cancer, with monarchE recently demonstrating a statistically significant OS benefit at 7 years (HR: 0.84, 95% CI: 0.72–0.98) [13,14]. Conversely, adjuvant palbociclib trials (PALLAS and PENELOPE-B) failed to demonstrate improvements in iDFS, underscoring heterogeneous efficacy in the adjuvant setting compared with MBC [15,16].

We conducted a systematic review and meta-analysis of EBC phase III trials to evaluate CDK4/6 inhibitor efficacy. We also analyzed MBC trials to quantify the magnitude of efficacy and treatment-related toxicity, thereby contextualizing the overall benefit–risk profile across disease stages.

## 2. Materials and Methods

### 2.1. Protocol Registration and Reporting Standards

This systematic review and meta-analysis was prospectively registered in PROSPERO (ID: CRD420251132302) and conducted in accordance with the Preferred Reporting Items for Systematic Reviews and Meta-Analyses (PRISMA) 2020 guidelines [17]. PRISMA 2020 checklist and checklists for abstracts are provided in the Supplementary Materials (Tables S1 and S2). The full protocol is available at https://www.crd.york.ac.uk/PROSPERO/view/CRD420251132302 (accessed on 21 January 2026).

### 2.2. Literature Search Strategy

A comprehensive search was conducted in MEDLINE (via PubMed), Embase, and Web of Science from inception to 1 October 2025, with assistance from a medical librarian. Search terms included MeSH (medical subject headings) and free-text terms for HR+/HER2− breast cancer, CDK4/6 inhibitors (palbociclib, abemaciclib, and ribociclib), and key trial names (MONARCH, PALOMA, MONALEESA, NATALEE, DAWNA, PATINA, EMERALD, and SONIA), combined using Boolean operators. Full search strategies are provided in Table S3. Results were imported into Covidence for deduplication, screening, and data management.

### 2.3. Study Selection

Two reviewers (R.D. and S.T.) independently screened titles, abstracts, and full texts; disagreements were resolved by consensus.

### 2.4. Inclusion Criteria

HR+/HER2− breast cancer (early or metastatic); phase III randomized controlled trials (RCTs) of CDK4/6 inhibitors plus ET; human participants; English-language full text.

### 2.5. Exclusion Criteria

Observational studies; case reports/series; editorials; letters; narrative reviews; phase I/II trials; studies including HER2+ or triple-negative populations, preclinical/animal studies; non-English publications.

### 2.6. Data Extraction

Two reviewers independently extracted data using standardized Covidence forms, including study characteristics, patient demographics, disease stage, interventions, follow-up, efficacy outcomes—OS, PFS, iDFS, DRFS (distant relapse-free survival)—ORR (objective response rate), CBR (clinical benefit rate), DCR (disease control rate), and toxicity endpoints (grade 3–4 adverse events), treatment discontinuation rate (TDR), and dose reduction rate (DRR). For EBC, pooled toxicity data were obtained from original trial reports [18,19,20], as recent updates did not report complete toxicity information [13,15,16].

### 2.7. Risk of Bias Assessment

Risk of bias was independently assessed using the Cochrane Risk of Bias 2.0 Tool, with disagreements resolved by consensus.

### 2.8. Statistical Analysis

Meta-analyses were performed using R software 4.5.2 (meta and metafor packages). Time-to-event outcomes (OS, PFS, iDFS, and DRFS) were pooled as hazard ratios (HRs) with 95% confidence intervals (CIs); dichotomous outcomes (ORR, CBR, DCR, TDR, DRR, and grade 3–4 adverse events) were pooled as risk ratios (RRs) with 95% CIs. Random-effects models with Hartung–Knapp adjustment were used for all pooled analyses. For outcomes reported by a single study, fixed-effects models were applied. Between-study heterogeneity was assessed using the Cochran Q, τ^2^, I^2^ statistics; I^2^ values exceeding 50% were considered indicative of substantial heterogeneity. Leave-one-out sensitivity analyses and funnel plots (≥10 studies) assessed the robustness and the publication bias, respectively.

## 3. Results

### 3.1. Database Search

**Figure 1:**
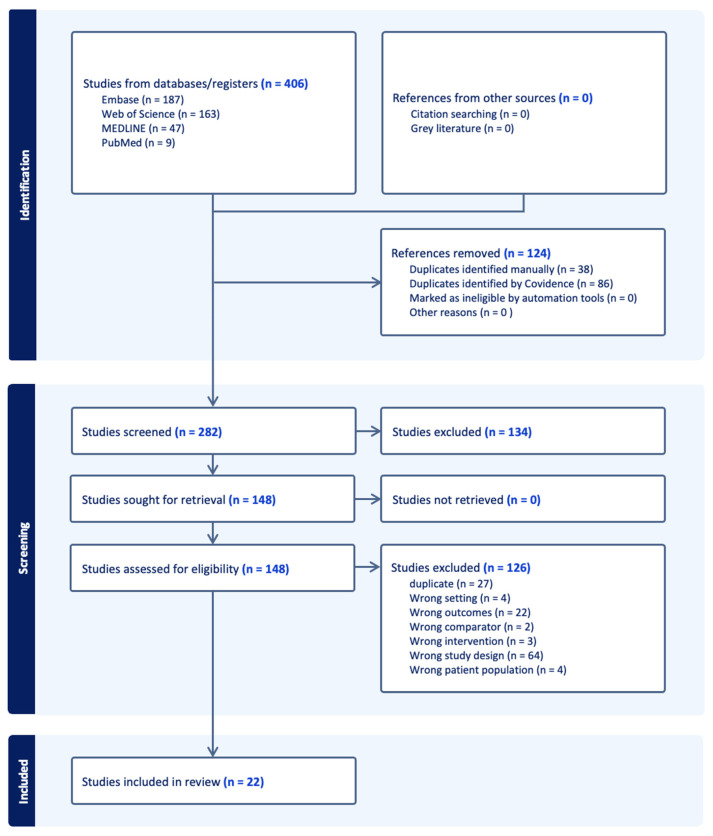
Preferred reporting items for systematic reviews and meta-analyses (PRISMA) 2020 flow diagram.

A total of 406 records were identified (Embase, 187; Web of Science, 163; MEDLINE, 47; PubMed, 9). After removing 124 duplicates, 282 records were screened; 134 were excluded after title and abstract screening due to an irrelevant study population, non-CDK4/6 inhibitor interventions, non-phase III study design, lack of relevant outcomes, or non-original article types (e.g., reviews, editorials, or commentaries) (Figure 1). One study (Hu 2025) [21] contributed two independently randomized cohorts, each analyzed separately, with its own control and treatment arm.

### 3.2. Study Characteristics

All included studies were RCTs evaluating CDK4/6 inhibitors (palbociclib, abemaciclib, ribociclib, dalpiciclib, and lerociclib) plus ET versus ET alone in HR+/HER2− breast cancer, across EBC and MBC. Key study characteristics are summarized in Table 1 [13,14,15,16,21,22,23,24,25,26,27,28,29,30,31,32,33,34,35,36,37,38].

### 3.3. Efficacy Outcomes

#### 3.3.1. Overall Survival (OS)

**Figure 2:**
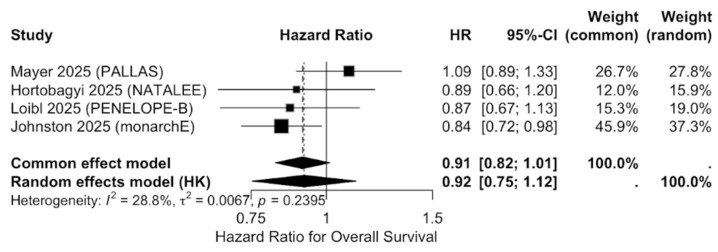
Forest plot of pooled HRs and 95% CIs for overall survival in early-stage disease [13,14,15,16]. Abbreviations: HR, hazard ratio; CI, confidence interval.

In EBC, CDK4/6 inhibitors did not significantly improve OS (HR: 0.92, 95% CI: 0.75–1.12; *p* = 0.40; Figure 2) with findings robust to leave-one-out sensitivity analyses (Figure S1).

**Figure 3:**
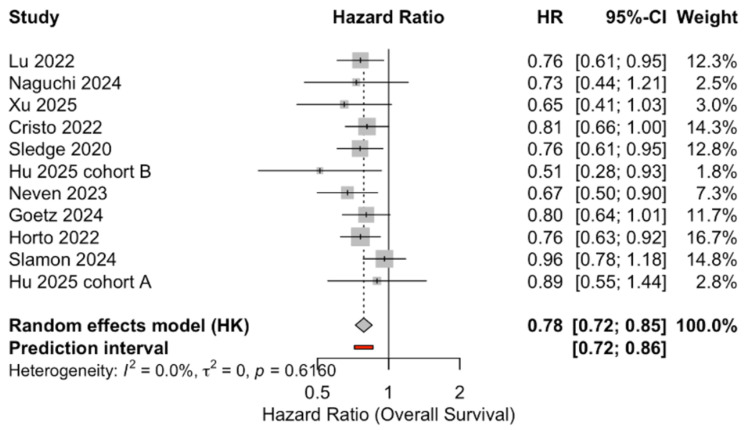
Forest plot of pooled HRs and 95% CIs for overall survival in metastatic disease [21,23,25,27,29,30,34,36,37,38].

**Figure 4:**
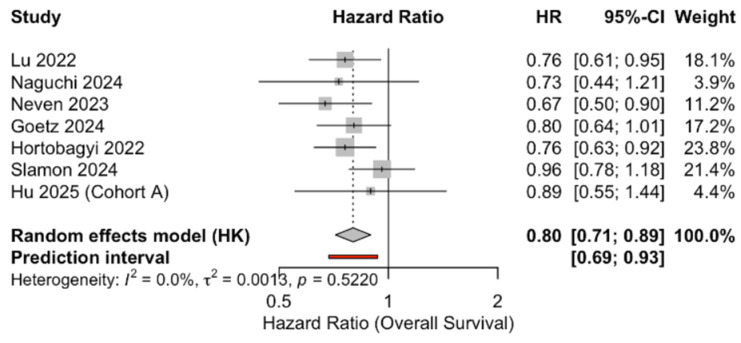
Forest plot of pooled HRs and 95% CIs for overall survival in the endocrine therapy-naive subgroup of metastatic disease [21,25,27,29,30,37,38].

**Figure 5:**
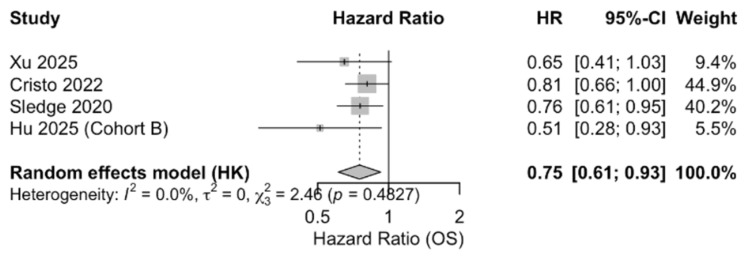
Forest plot of pooled HRs and 95% CIs for overall survival in patients with metastatic disease who progressed on endocrine therapy (mixed menopausal population) [21,23,34,36].

**Figure 6:**
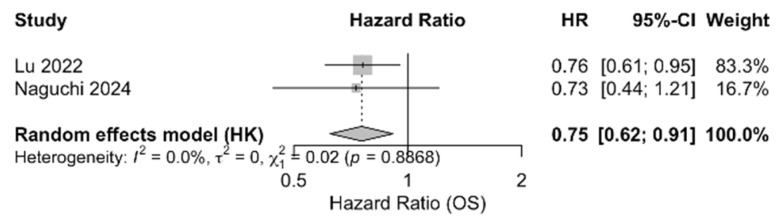
Forest plot of pooled HRs and 95% CIs for overall survival in pre- and peri-menopausal subgroup of metastatic disease [29,37].

**Figure 7:**
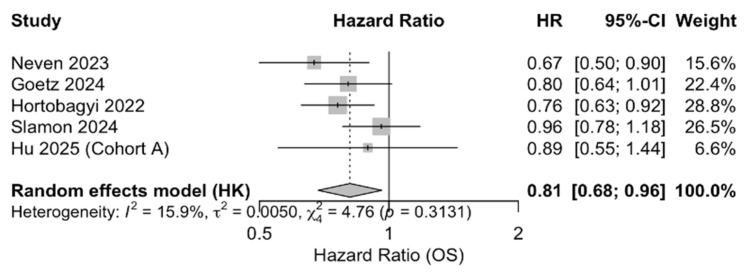
Forest plot of pooled HRs and 95% CIs for overall survival in post-menopausal subgroup of metastatic disease [21,25,27,30,38].

In MBC, OS was significantly improved (HR: 0.78; 95% CI: 0.72–0.85; *p* < 0.001; Figure 3), with negligible heterogeneity. Leave-one-out sensitivity analyses demonstrated that OS findings were robust to the exclusion of individual studies (Figure S2), and visual inspection of funnel plots showed no marked asymmetry, suggesting low likelihood of publication bias (Figure S3). Subgroup analyses demonstrated consistent OS benefit irrespective of prior ET exposure (ET-naive: HR: 0.80, 95% CI: 0.71–0.89, *p* < 0.001, Figure 4; previously ET-treated: HR: 0.75, 95% CI: 0.61–0.93, *p* = 0.009, Figure 5), and menopausal status (Figure 6 and Figure 7).

#### 3.3.2. Progression-Free Survival (PFS)

**Figure 8:**
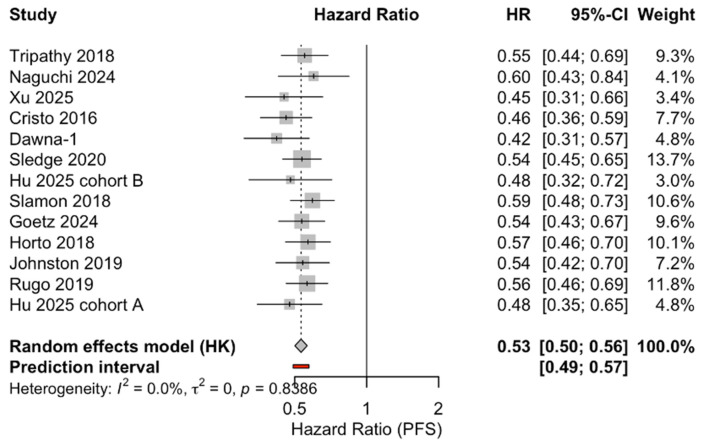
Forest plot of pooled HRs and 95% CIs for progression-free survival in metastatic disease [21,22,24,25,26,28,31,32,34,35,36,37].

**Figure 9:**
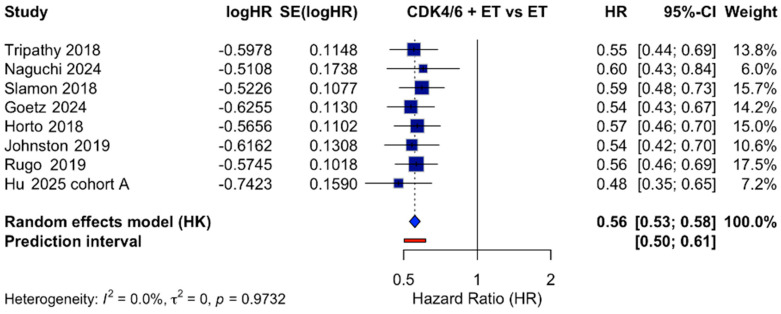
Forest plot of pooled HRs and 95% CIs for progression-free survival in endocrine therapy-naive subgroup of metastatic disease [21,25,26,28,31,32,35,37].

**Figure 10:**
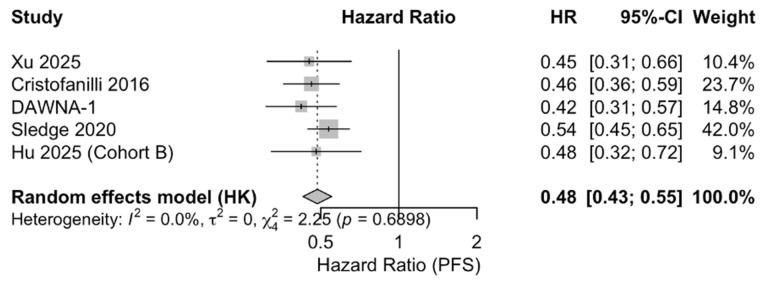
Forest plot of pooled HRs and 95% CIs for progression-free survival in metastatic disease patients who progressed on endocrine therapy [21,22,24,34,36].

In MBC, PFS was significantly improved (HR: 0.53, 95% CI: 0.50–0.56; *p* < 0.001; Figure 8), with consistent benefit across ET lines (Figure 9 and Figure 10) and menopausal subgroups (pre-/peri-menopausal: HR: 0.56, 95% CI: 0.34–0.94, I^2^ = 0%; post-menopausal: HR: 0.55, 95% CI: 0.52–0.59, I^2^ = 0%). PFS was not reported in EBC trials.

#### 3.3.3. Invasive Disease-Free Survival (iDFS) and Distant Relapse-Free Survival (DRFS)

**Figure 11:**
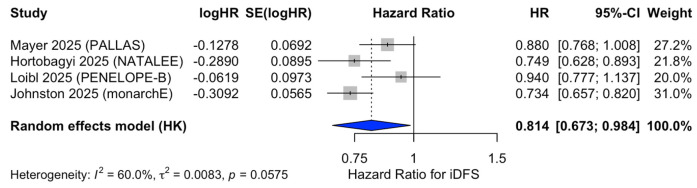
Forest plot of pooled HRs and 95% CIs for invasive disease-free survival in early-stage disease [13,14,15,16].

In EBC, iDFS was significantly improved (HR: 0.81, 95% CI: 0.67–0.98; *p* = 0.029; I^2^ = 60%; Figure 11), while DRFS showed a non-significant trend (HR: 0.81; 95% CI: 0.24–2.67; *p* = 0.73; I^2^ = 73.2%). These endpoints were not reported in MBC trials.

#### 3.3.4. Response Rates

In MBC, CDK4/6 inhibitors improved ORR (RR: 2.07; 95% CI: 1.26–3.40; *p* = 0.004; I^2^ = 92.5%), with consistent benefit in ET-naive (RR: 1.51, 95% CI: 1.34–1.71; *p* < 0.001; I^2^ = 0%) and previously ET-treated patients (RR: 2.12, 95% CI: 1.15–3.89; *p* = 0.016; I^2^ = 0.5%). CBR was improved overall (RR: 1.25; 95% CI: 1.18–1.32; *p* < 0.001; I^2^ = 72.9%), particularly in ET-naive patients (RR: 1.43; 95% CI: 1.14–1.78; *p* = 0.002), with no significant improvement in previously ET-treated patients (RR: 1.15, 95% CI: 0.91–1.46, *p* = 0.24). DCR was significantly improved (RR: 1.09; 95% CI: 1.04–1.15; *p* = 0.001; I^2^ = 57.1%) with greater benefit in previously ET-treated patients (RR: 1.14, 95% CI: 1.06–1.22; *p* = 0.002; I^2^ = 0.4%). These endpoints were not reported in EBC trials.

### 3.4. Toxicity Outcomes

#### 3.4.1. Treatment Modifications

In EBC, TDR (RR: 8.39, 95% CI: 2.24–31.42; *p* = 0.014; I^2^ = 95.7%) and DRR (RR: 26.84, 95% CI: 14.85–48.49; *p* < 0.0001) were markedly increased. In MBC, TDR (RR: 2.39; 95% CI: 1.54–3.70; *p* < 0.001; I^2^ = 48.2%) and DRR (RR: 9.51; 95% CI: 6.96–13.01; *p* < 0.001; I^2^ = 27.5%) were also significantly elevated.

#### 3.4.2. Individual Grade 3–4 Adverse Events

Individual grade 3–4 adverse events were evaluated for hematologic and non-hematologic toxicities in EBC and MBC trials (Tables S4 and S5). Neutropenia was the most frequent high-grade hematologic toxicity in both disease settings, while leukopenia, anemia, and thrombocytopenia occurred at more modest but elevated rates. In MBC trials, diarrhea, hepatic transaminase elevations, and infections were significantly increased compared with ET alone, whereas in EBC trials, fatigue and hepatic transaminase elevations were most pronounced.

**Figure 12:**
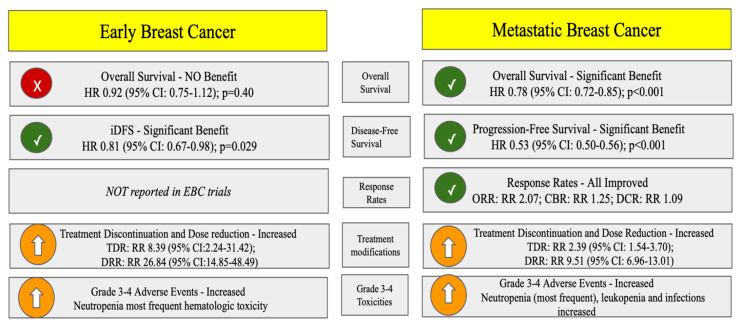
Schematic summary of pooled efficacy and safety outcomes of CDK4/6 inhibitors in HR+/HER2- breast cancer. Abbreviations: iDFS, invasive disease-free survival; TDR, treatment discontinuation rate; DRR, dose reduction rate; ORR, objective response rate; CBR, clinical benefit rate; DCR, disease control rate. Red circle: No benefit; Green circle: Significant benefit; Orange circle: Increased benefit.

A comprehensive summary of all pooled efficacy and toxicity outcomes with corresponding hazard ratios, risk ratios, and confidence intervals is presented in Figure 12.

### 3.5. Risk of Bias Assessment

Most trials were judged at low risk of bias. PALOMA-2 and its extended follow-up had a high risk of bias; PALOMA-3 (OS update), NATALEE, monarchE (final OS analysis), and PALLAS had some concerns (Figure S4).

## 4. Discussion

CDK4/6 inhibitors demonstrated distinct efficacy profiles in EBC and MBC. In MBC, CDK4/6 inhibitor-based therapy was associated with substantial and consistent improvements in OS (HR: 0.78, 95% CI: 0.72–0.85) and PFS (HR: 0.53, 95% CI: 0.50–0.56), with minimal heterogeneity. Although TDR and DRR were increased in MBC, driven by predictable class-related toxicities such as hematologic, gastrointestinal, hepatic, and infectious adverse events, these were largely manageable and did not attenuate the survival benefits [39,40,41]. In contrast, in EBC, CDK4/6 inhibitors significantly improved iDFS (HR: 0.81, 95% CI: 0.67–0.98) but did not confer a statistically significant pooled OS benefit (HR: 0.92, 95% CI: 0.75–1.12). This supports a favorable benefit–risk profile in MBC while highlighting a more nuanced therapeutic role in EBC.

These results are consistent with prior meta-analyses demonstrating durable survival benefits with CDK4/6 inhibitors in MBC and improvements in recurrence-related outcomes without a clear OS benefit in EBC [42,43,44,45]. Our analysis extends the literature by incorporating the most recent data, systematically integrating toxicity outcomes, and enabling stage-specific interpretation, providing a more comprehensive benefit–risk assessment.

The monarchE trial recently reported the first statistically significant OS benefit among adjuvant CDK4/6 inhibitor trials, with follow-up demonstrating 7-year OS of 86.8%versus 85% (HR: 0.842; 95% CI: 0.722–0.981), an absolute difference of 1.8% [13]. While this validates that iDFS improvements may translate into survival gains with sufficient follow-up, the OS benefit remains modest relative to iDFS [13]. Our pooled analysis, which did not demonstrate OS benefit in EBC, likely reflects data immaturity, as follow-up in NATALEE, PALLAS, and PENELOPE-B remains shorter than in monarchE [13,14,15,16]. Additionally, effective salvage therapies and competing causes of death may attenuate observable survival differences in the adjuvant setting [46,47].

Heterogeneity across adjuvant trials can be partially explained by differences in patient selection, treatment duration, and drug-specific characteristics [13,14,15,16,48]. MonarchE enrolled a strictly high-risk population, PALLAS included a broader intermediate-risk cohort, and NATALEE enrolled stage IIB-IIIC patients and selected node-negative patients with additional high-risk features (large tumor size, high grade, or elevated Ki-67) [13,14,15]. Collectively, these differences in baseline risk and eligibility criteria likely contributed to the variable magnitude of benefit observed across adjuvant CDK4/6 inhibitor trials. Treatment duration varied across the adjuvant trials, with ribociclib administered for 3 years in NATALEE, palbociclib for 2 years in PALLAS, 1 year in PENELOPE-B, and abemaciclib for 2 years in monarchE, which may have contributed to differences in outcomes [13,14,15,16]. Pharmacologic differences may contribute to heterogeneity: abemaciclib and ribociclib show greater CDK4 versus CDK6 selectivity, with abemaciclib uniquely inhibiting other kinases and delivered continuously, in contrast to the intermittent dosing of palbociclib and ribociclib [13,14,15,48]. Notably, both positive adjuvant trials (monarchE and NATALEE) employed open-label designs, raising the possibility of informative censoring and bias [13,14].

High rates of treatment discontinuation represent a major challenge in the adjuvant setting and may attenuate therapeutic benefit [49]. Our pooled analysis of EBC demonstrated markedly increased TDR (RR: 8.39, 95% CI: 2.24–31.42; *p* = 0.014) and DRR (RR: 26.84, 95% CI: 14.85–48.49, *p* < 0.001), with fatigue and hepatic transaminase elevations being the most pronounced adverse events. These findings are consistent with observations from individual trials, including PALLAS (42% discontinuations), monarchE and NATALEE, where discontinuations were common and primarily driven by adverse events [13,14,15]. Patient-related factors such as older age, comorbidity burden, and reduced functional status have been associated with higher discontinuation risk [50]. Reduced cumulative drug exposure may mask the true efficacy of adjuvant CDK4/6 inhibition, underscoring the importance of careful patient selection and proactive toxicity management.

Biological differences between EBC and MBC may further explain the divergence in survival benefit [50,51,52,53,54]. MBC is characterized by clonal evolution and sustained dependence on proliferative signaling through the cyclin D-CDK4/6-Rb axis, rendering tumors particularly susceptible to continuous CDK4/6 inhibition [51,52,53]. In contrast, EBC encompasses heterogeneous and often dormant tumor cell populations, including disseminated tumor cells that may evade therapies targeting active cell-cycle progression [54,55]. The fixed duration (2–3 years) of adjuvant CDK4/6 inhibitor therapy may be insufficient to eradicate slow-cycling or dormant cells, which may re-enter the cell cycle following treatment cessation [13,14,15,16,56,57]. Moreover, CDK4/6 inhibitors appear to induce cellular senescence rather than permanent elimination of tumor cells, and senescent cells may regain proliferative capacity after drug withdrawal [58,59,60]. In contrast, in MBC, continuous administration of CDK4/6 inhibitors suppresses actively proliferating tumor clones over time, which provides a plausible mechanistic explanation for the observed benefit in RCTs [23,24,25,29].

In addition to these cell-cycle-dependent mechanisms, CDK4/6 inhibitors exert immunomodulatory effects, including enhanced tumor antigen presentation and suppression of regulatory T-cell proliferation [61]. These effects may be more relevant in MBC, where tumor burden and immune engagement are greater, whereas their clinical significance in the adjuvant setting with minimal residual disease remains uncertain.

Beyond differential immune engagement, resistance mechanisms may also differ between EBC and MBC settings. Resistance mechanisms such as RB1 loss, CCNE1 amplification, and activation of the PI3K/AKT/mTOR pathway have been clinically established in MBC through genomic sequencing of resistant tumors [62,63]. In contrast, the biological landscape of resistance in the adjuvant setting remains poorly characterized. A small, hypothesis-generating abstract of patients relapsing on adjuvant abemaciclib (n = 10) reported frequent TP53 pathway alterations (90%) and loss of estrogen receptor expression (50%) but notably no RB1 mutations, suggesting potentially distinct mechanisms from MBC [64]. Larger prospective studies are needed to validate these findings and further define resistance pathways in the adjuvant setting.

While CDK4/6 inhibitor-related toxicities are manageable in MBC [39,40,41], tolerability is more consequential in the adjuvant setting [19], where long-term survival benefit has not been clearly established [13,14]. In adjuvant trials, the health-related quality of life was largely preserved but not improved, and treatment was associated with increased toxicity and financial burden [65]. Consistent with this, cost-effectiveness analyses demonstrated that adjuvant CDK4/6 inhibitor therapy exceeds commonly accepted willingness-to-pay thresholds [66,67]; for example, ribociclib plus ET was estimated to have an incremental cost-effectiveness ratio of approximately $1.2 million per equal value life year gained, requiring an estimated 90% price reduction to meet accepted benchmarks [66]. These considerations are particularly important in a largely curable population, where even modest absolute gains—such as the ~1.8% improvement in OS at 7 years with adjuvant abemaciclib in monarchE [13]—must be weighed against cumulative toxicity, treatment burden, and cost.

Despite these economic and tolerability concerns, current National Comprehensive Cancer Network (NCCN) and American Society of Clinical Oncology (ASCO) guidelines recommend adjuvant CDK4/6 inhibitors for selected patients with HR+/HER2− EBC, based on significant improvements in iDFS observed in RCTs [68,69]. Abemaciclib is recommended for 2 years in combination with ET for high-risk, node-positive patients (≥4 positive lymph nodes or 1–3 positive nodes with grade 3 disease, tumor size ≥5 cm, or Ki-67 ≥20%) [68,69]. In contrast, ribociclib is recommended for 3 years in combination with an aromatase inhibitor in a broader population, including patients with non-microscopic nodal involvement or selected node-negative patients with high-risk features [68]. ASCO further emphasizes that adjuvant CDK4/6 inhibitors may not provide meaningful benefit for all trial-eligible patients, particularly lower-risk subgroups in NATALEE, and recommends individualized decision-making that incorporates expected benefit, toxicity, cost, and patient preferences [69].

The strengths of this paper are the inclusion of only phase III randomized controlled trials with a large pooled population, which enhanced statistical power to detect clinically meaningful differences in both efficacy and toxicity outcomes. Additionally, conducting separate analyses for EBC and MBC enabled context-specific interpretation of results across disease settings. The use of a random-effects model with Hartung–Knapp adjustment further strengthened the robustness of the findings by appropriately accounting for between-study heterogeneity. The limitations were the trial-level design, which precluded individual patient-level analyses and restricted more granular subgroup exploration, as well as the relatively shorter follow-up duration in EBC trials, leading to immature OS data.

## 5. Conclusions

This meta-analysis of phase III RCTs demonstrates a clear OS benefit for CDK4/6 inhibitors in MBC, supporting their established role in this setting. In EBC, CDK4/6 inhibitors consistently improve recurrence-related outcomes, but survival benefit remains limited at the meta-analytic level, despite emerging trial-level signals from monarchE. In the adjuvant setting, these gains must be carefully weighed against treatment-related toxicity, financial burden, and patient-specific factors. Future studies should aim to optimize the benefit–risk balance and cost-effectiveness, with extended follow-up to clarify long-term outcomes in the adjuvant setting.

## Figures and Tables

**Table 1:** Characteristics of included phase III randomized trials of CDK4/6 inhibitors in HR+/HER2− breast cancer.

Author, Year, Trial	Study Design	Disease Stage	Sample Size (TA/CA)	Prior ET Status	Intervention	Comparator	Median Follow-Up	Endpoints Extracted for Analysis
Hortobagyi 2025 (NATALEE) [14]	Open label	Early	2549/2552	Naive	Ribociclib + ET	ET	33.3 months	OS, iDFS
Loibl 2025 (PENELOPE-B) [16]	Double-blind	Early	631/619	Naive	Palbociclib + ET	Placebo	77.8 months	OS, iDFS
Mayer 2025 (PALLAS) [15]	Open label	Early	2883/2870	Naive	Palbociclib + ET	ET	59.8 months	OS, iDFS, DRFS
Johnston 2025 (monarchE) [13]	Open label	Early	2808/2829	Naive	Abemaciclib + ET	ET	76.2 months	OS, iDFS, DRFS
Cristofanilli 2016 (PALOMA-3) [22]	Double-blind	Metastatic	347/174	Progressed on ET	Palbociclib + Fulvestrant	Fulvestrant + Placebo	8.9 months	PFS, CBR, TDR, DRR
Cristofanilli 2022 (PALOMA-3) [23]	Double-blind	Metastatic	347/174	Progressed on ET	Palbociclib + Fulvestrant	Fulvestrant + Placebo	73.3 months	OS, safety
Xu 2021 (DAWNA-1) [24]	Double-blind	Metastatic	241/120	Progressed on ET	Dalpiciclib + Fulvestrant	Fulvestrant + Placebo	10.7 months	PFS, ORR, CBR, TDR, DRR, safety
Goetz 2024 (MONARCH 3) [25]	Double-blind	Metastatic	328/165	Naive	Abemaciclib + ET	ET	8.1 years	PFS, OS, safety
Hortobagyi 2018 (MONALEESA-2) [26]	Double-blind	Metastatic	334/334	Naive	Ribociclib + ET	Placebo + ET	26.4 months	PFS, TDR, DRR
Hortobagyi 2022 (MONALEESA-2) [27]	Double-blind	Metastatic	334/334	Naive	Ribociclib + ET	Placebo + ET	6.6 years	OS, safety
Hu 2025 cohort A (MONARCH plus) [21]	Double-blind	Metastatic	207/99	Naive	Abemaciclib + ET	Placebo + ET	30.1 months	PFS, OS, ORR, CBR, DCR, TDR, Safety
Hu 2025 cohort B (MONARCH plus) [21]	Double-blind	Metastatic	104/53	Progressed on ET	Abemaciclib + ET	Placebo + ET	26 months	PFS, OS, ORR, CBR, DCR, TDR, safety
Johnston 2019 (MONARCH 3) [28]	Double-blind	Metastatic	328/165	Naive	Abemaciclib + ET	Placebo + ET	26.7 months	ORR, CBR, DCR
Lu 2022 (MONALEESA-7) [29]	Double-blind	Metastatic	335/337	Naive	Ribociclib + ET	Placebo + ET	53.5 months	OS, safety
Neven 2023 (MONALEESA-3) [30]	Double-blind	Metastatic	484/242	Naive	Ribociclib + ET	Placebo + ET	70.8 months	OS, safety
Rugo 2019 (PALOMA-2) [31]	Double-blind	Metastatic	444/222	Naive	Palbociclib + ET	Placebo + ET	38 months	PFS
Slamon 2018 (MONALEESA-3) [32]	Double-blind	Metastatic	484/242	Naive	Ribociclib + ET	Placebo + ET	56.3 months	PFS, ORR, CBR
Sledge 2017 (MONARCH 2) [33]	Double-blind	Metastatic	446/223	Progressed on ET	Abemaciclib + ET	Placebo + ET	19.5 months	ORR, CBR, DCR
Sledge 2020 (MONARCH 2) [34]	Double-blind	Metastatic	446/223	Progressed on ET	Abemaciclib + ET	Placebo + ET	47.7 months	PFS, OS, safety
Tripathy 2018 (MONALEESA-7) [35]	Double-blind	Metastatic	335/337	Naive	Ribociclib + ET	Placebo + ET	19.2 months	PFS, TDR, DRR
Xu 2025 (LEONARDA-1) [36]	Double-blind	Metastatic	137/138	Progressed on ET	Lerociclib + ET	Placebo + ET	7.36 months	PFS, OS, ORR, CBR, DCR, TDR, DRR, safety
Noguchi 2024 (PATHWAY) [37]	Double-blind	Metastatic	91/93	Naive	Palbociclib + ET	Placebo + ET	40.9 months	PFS, OS, ORR, CBR, safety
Slamon 2024 (PALOMA-2) [38]	Double-blind	Metastatic	444/222	Naive	Palbociclib + ET	Placebo + ET	90.1 months	OS, safety

Abbreviations: TA, treatment arm; CA, control arm; ET, endocrine therapy; OS, overall survival; iDFS, invasive disease-free survival; DRFS, distant relapse-free survival; PFS, progression-free survival; ORR, objective response rate; CBR, clinical benefit rate; DCR, disease control rate; TDR, treatment discontinuation rate; DRR, dose reduction rate.

## Data Availability

No new data were created or analyzed in this study. Data sharing is not applicable to this article.

## Supplementary Materials

The following supporting information can be downloaded at https://www.mdpi.com/article/10.3390/cancers18111714/s1, Table S1: PRISMA 2020 Main Checklist; Table S2: PRISMA 2020 Checklists for Abstracts; Table S3: Search Strategy for Phase III Cyclin-Dependent Kinase 4/6 (CDK4/6) Inhibitor Trials in Hormone Receptor-Positive/Human Epidermal Growth Factor Receptor 2– (HR+/HER2−) Breast Cancer; Table S4: Hematologic Adverse Events in Early- and Metastatic-Stage Disease; Table S5: Non-hematological Adverse Events in Early- and Metastatic-Stage Disease; Figure S1: Leave-One-Out Sensitivity Analysis for Overall Survival (OS) in Early-Stage Disease; Figure S2: Leave-One-Out Sensitivity Analysis for Overall Survival (OS) in Metastatic Disease; Figure S3: Funnel Plot of Overall Survival (OS) in Metastatic Disease; Figure S4: Risk of Bias Assessment in Randomized-Controlled Trials.

## Abbreviations

The following abbreviations are used in this manuscript:

HER2	Human epidermal growth factor receptor 2
HR+	Hormone receptor-positive
HER2−	Human epidermal growth factor receptor 2-negative
EBC	Early-stage breast cancer
MBC	Metastatic breast cancer
ET	Endocrine therapy
CDK4/6	Cyclin D–cyclin-dependent kinase 4 and 6
Rb	Retinoblastoma protein
PFS	Progression-free survival
OS	Overall survival
iDFS	Invasive disease-free survival
HR	Hazard ratio
CI	Confidence interval
PRISMA	Preferred Reporting Items for Systematic Reviews and Meta-Analyses
MeSH	Medical subject headings
DRFS	Distant relapse-free survival
ORR	Objective response rate
CBR	Clinical benefit rate
DCR	Disease control rate
TDR	Treatment discontinuation rate
DRR	Dose reduction rate
RCTs	Randomized control trials
TA	Treatment arm
CA	Control arm
NCCN	National Comprehensive Cancer Network
ASCO	American Society of Clinical Oncology
