# Supplementary material for: Efficacy and Toxicity of CDK4/6 Inhibitors in Early and Metastatic HR+/HER2− Breast Cancer: An Updated Meta-Analysis of Phase III Trials
Source: Cancers (Basel). 2026 May 24;18(11):1714. doi: 10.3390/cancers18111714 (PMC13255879; doi:10.3390/cancers18111714)
Supplement: Supplementary file 1 [file cancers-18-01714-s001.zip › cancers-4188457-supplementary.pdf]

Supplements

Table S1. PRISMA 2020 Main Checklist

Topic	No.	Item	Location where item is reported
**TITLE**			
**Title**	1	Identify the report as a systematic review.	Title page
**ABSTRACT**			
**Abstract**	2	See the PRISMA 2020 for Abstracts checklist	Abstract section
**INTRODUCTION**			
**Rationale**	3	Describe the rationale for the review in the context of existing knowledge.	1. Introduction, paragraphs 1–3
**Objectives**	4	Provide an explicit statement of the objective(s) or question(s) the review addresses.	1. Introduction, final paragraph
**METHODS**			
**Eligibility criteria**	5	Specify the inclusion and exclusion criteria for the review and how studies were grouped for the syntheses.	2. Materials and Methods, Study Selection (Inclusion and exclusion criteria)
**Information sources**	6	Specify all databases, registers, websites, organisations, reference lists and other sources searched or consulted to identify studies. Specify the date when each source was last searched or consulted.	2. Materials and Methods, Literature Search Strategy
**Search strategy**	7	Present the full search strategies for all databases, registers and websites, including any filters and limits used.	2. Materials and Methods, Literature Search Strategy, Supplementary materials (Table S3)

Topic	No.	Item	Location where item is reported
**Selection process**	8	Specify the methods used to decide whether a study met the inclusion criteria of the review, including how many reviewers screened each record and each report retrieved, whether they worked independently, and if applicable, details of automation tools used in the process.	2. Materials and Methods, Study Selection
**Data collection process**	9	Specify the methods used to collect data from reports, including how many reviewers collected data from each report, whether they worked independently, any processes for obtaining or confirming data from study investigators, and if applicable, details of automation tools used in the process.	2. Materials and Methods, Data Extraction
**Data items**	10a	List and define all outcomes for which data were sought. Specify whether all results that were compatible with each outcome domain in each study were sought (e.g. for all measures, time points, analyses), and if not, the methods used to decide which results to collect.	2. Materials and Methods, Data Extraction
	10b	List and define all other variables for which data were sought (e.g. participant and intervention characteristics, funding sources). Describe any assumptions made about any missing or unclear information.	2. Materials and Methods, Data Extraction

Topic	No.	Item	Location where item is reported
**Study risk of bias assessment**	11	Specify the methods used to assess risk of bias in the included studies, including details of the tool(s) used, how many reviewers assessed each study and whether they worked independently, and if applicable, details of automation tools used in the process.	2. Materials and Methods, Risk of Bias Assessment
**Effect measures**	12	Specify for each outcome the effect measure(s) (e.g. risk ratio, mean difference) used in the synthesis or presentation of results.	2. Materials and Methods, Statistical Analysis
**Synthesis methods**	13a	Describe the processes used to decide which studies were eligible for each synthesis (e.g. tabulating the study intervention characteristics and comparing against the planned groups for each synthesis (item 5)).	2. Materials and Methods, Study selection and Data Extraction
	13b	Describe any methods required to prepare the data for presentation or synthesis, such as handling of missing summary statistics, or data conversions.	2. Materials and Methods, Data Extraction and Statistical Analysis
	13c	Describe any methods used to tabulate or visually display results of individual studies and syntheses.	2. Materials and Methods, Statistical Analysis

Topic	No.	Item	Location where item is reported
	13d	Describe any methods used to synthesize results and provide a rationale for the choice(s). If meta-analysis was performed, describe the model(s), method(s) to identify the presence and extent of statistical heterogeneity, and software package(s) used.	2. Materials and Methods, Statistical Analysis
	13e	Describe any methods used to explore possible causes of heterogeneity among study results (e.g. subgroup analysis, meta-regression).	2. Materials and Methods, Statistical Analysis
	13f	Describe any sensitivity analyses conducted to assess robustness of the synthesized results.	2. Materials and Methods, Statistical Analysis
**Reporting bias assessment**	14	Describe any methods used to assess risk of bias due to missing results in a synthesis (arising from reporting biases).	2. Materials and Methods, Statistical Analysis
**Certainty assessment**	15	Describe any methods used to assess certainty (or confidence) in the body of evidence for an outcome.	Not performed
**RESULTS**			
**Study selection**	16a	Describe the results of the search and selection process, from the number of records identified in the search to the number of studies included in the review, ideally using a flow diagram.	3.1 Database search; Figure 1 (PRISMA flow diagram)
	16b	Cite studies that might appear to meet the inclusion criteria, but which were excluded, and explain why they were excluded.	3.1 Database search

Topic	No.	Item	Location where item is reported
**Study characteristics**	17	Cite each included study and present its characteristics.	3.2 Study characteristics; Table 1
**Risk of bias in studies**	18	Present assessments of risk of bias for each included study.	3.5 Risk of Bias Assessment; Figure S4
**Results of individual studies**	19	For all outcomes, present, for each study: (a) summary statistics for each group (where appropriate) and (b) an effect estimate and its precision (e.g. confidence/credible interval), ideally using structured tables or plots.	3.3 Efficacy outcomes; 3.4 Toxicity outcomes; Forest plots (Figures 2-11)<br>Table S4-5
**Results of syntheses**	20a	For each synthesis, briefly summarise the characteristics and risk of bias among contributing studies.	3.2 Study characteristics, and 3.5 Risk of bias Assessment,Table 1; Figure S4
	20b	Present results of all statistical syntheses conducted. If meta-analysis was done, present for each the summary estimate and its precision (e.g. confidence/credible interval) and measures of statistical heterogeneity. If comparing groups, describe the direction of the effect.	3.3 Efficacy outcomes, and 3.4 Toxicity outcomes; Figures 2–11; Tables S4–5)
	20c	Present results of all investigations of possible causes of heterogeneity among study results.	3.3 Efficacy outcomes, and 3.4 Toxicity outcomes
	20d	Present results of all sensitivity analyses conducted to assess the robustness of the synthesized results.	3.3 Efficacy outcomes and 3.4 Toxicity outcomes; Supplement (Figures S1–2)

Topic	No.	Item	Location where item is reported
**Reporting biases**	21	Present assessments of risk of bias due to missing results (arising from reporting biases) for each synthesis assessed.	3.5 Risk of Bias Assessment; Figure S4
**Certainty of evidence**	22	Present assessments of certainty (or confidence) in the body of evidence for each outcome assessed.	Not assessed
**DISCUSSION**			
**Discussion**	23a	Provide a general interpretation of the results in the context of other evidence.	4. Discussion
	23b	Discuss any limitations of the evidence included in the review.	4. Discussion
	23c	Discuss any limitations of the review processes used.	4. Discussion
	23d	Discuss implications of the results for practice, policy, and future research.	4. Discussion
**OTHER INFORMATION**			
**Registration and protocol**	24a	Provide registration information for the review, including register name and registration number, or state that the review was not registered.	2. Materials and Methods, Protocol Registration and Reporting Standards
	24b	Indicate where the review protocol can be accessed, or state that a protocol was not prepared.	2. Materials andMethods, Protocol Registration and Reporting Standards
	24c	Describe and explain any amendments to information provided at registration or in the protocol.	2. Methods, Protocol Registration and Reporting Standards (PROSPERO record updated)

Topic	No.	Item	Location where item is reported
**Support**	25	Describe sources of financial or non-financial support for the review, and the role of the funders or sponsors in the review.	Funding section
**Competing interests**	26	Declare any competing interests of review authors.	Conflicts of Interest
**Availability of data, code and other materials**	27	Report which of the following are publicly available and where they can be found: template data collection forms; data extracted from included studies; data used for all analyses; analytic code; any other materials used in the review.	Data Availability Statement

Abbreviation: PRISMA, Preferred Reporting Items for Systematic reviews and Meta-Analyses.

Table S2. PRISMA 2020 Checklists for Abstracts

Topic	No.	Item	Reported?
**TITLE**			
**Title**	1	Identify the report as a systematic review.	Yes
**BACKGROUND**			
**Objectives**	2	Provide an explicit statement of the main objective(s) or question(s) the review addresses.	Yes
**METHODS**			
**Eligibility criteria**	3	Specify the inclusion and exclusion criteria for the review.	Yes
**Information sources**	4	Specify the information sources (e.g. databases, registers) used to identify studies and the date when each was last searched.	Yes
**Risk of bias**	5	Specify the methods used to assess risk of bias in the included studies.	Yes
**Synthesis of results**	6	Specify the methods used to present and synthesize results.	Yes
**RESULTS**			
**Included studies**	7	Give the total number of included studies and participants and summarise relevant characteristics of studies.	Yes

Topic	No.	Item	Reported?
**Synthesis of results**	8	Present results for main outcomes, preferably indicating the number of included studies and participants for each. If meta-analysis was done, report the summary estimate and confidence/credible interval. If comparing groups, indicate the direction of the effect (i.e. which group is favoured).	Yes
**DISCUSSION**			
**Limitations of evidence**	9	Provide a brief summary of the limitations of the evidence included in the review (e.g. study risk of bias, inconsistency and imprecision).	Yes
**Interpretation**	10	Provide a general interpretation of the results and important implications.	Yes
**OTHER**			
**Funding**	11	Specify the primary source of funding for the review.	Not reported
**Registration**	12	Provide the register name and registration number.	Yes

Abbreviation: PRISMA, Preferred Reporting Items for Systematic reviews and Meta-Analyses.

Table S3. Search Strategy for Phase III Cyclin-Dependent Kinase 4/6 (CDK4/6) Inhibitor Trials in Hormone Receptor-Positive/Human Epidermal Growth Factor Receptor 2– (HR+/HER2–) Breast Cancer

Step	Search strategy	Results
1	HR+$HER2- breast cancer.mp.	470
2	(("hormone*receptor positive" or HER2 negative) adj "breast cancer").m_titl.	520
3	1 or 2	948
4	(early or metastatic).m_titl.	500224
5	3 and 4	213
6	Cyclin-Dependent Kinase 4/ or Cyclin-Dependent Kinase 6/	5308
7	"CDK4/6 Inhibitors".mp.	1834
8	6 or 7	6289
9	5 and 8	37
10	limit 9 to english language	37
11	((MONARCH* or NATALEE or PALOMA or MONALEESA or DAWNA or PATINA or PATRIICA or EMERALD or SONIA) and breast cancer).m_titl.	137
12	10 or 11	174
13	("phase 3" or "phase III").mp.	83440
14	12 and 13	81
15	limit 14 to (english language and humans and clinical trial, phase iii)	48

Abbreviations: adj, adjacency operator; CDK4/6, cyclin-dependent kinase 4/6; HER2–, human epidermal growth factor receptor 2–negative; HR+, hormone receptor–positive; MeSH, Medical Subject Headings; mp, multipurpose field (title, abstract, subject headings, keywords); m_titl, title field.

[a]Symbols used in search strategy: / indicates a MeSH term or controlled vocabulary term; * is the truncation (wildcard) symbol; Boolean operator OR was used to combine synonyms, and AND was used to combine different conceptual domains.

[b]Database: MEDLINE (PubMed), Embase,and Web of Science; Search period: Inception to October 1, 2025.

Table S4. Hematologic Adverse Events in Early- and Metastatic-Stage Disease

Adverse Event	Grade	Disease Stage	No. of Trials	Pooled effect estimate (RR)	95% CI	I^2(%)	Model
Neutropenia	3	Early	4	58.74	16.96 - 203.43	89.2%	RE(HK)
Neutropenia	3	Metastatic	12	24.89	12.42-49.86	63.1%	RE(HK)
Neutropenia	4	Early	3	46.53	1.54-1398.09	43.8%	RE(HK)
Neutropenia	4	Metastatic	11	11.87	4.31-32.71	39.9%	RE(HK)
Leukopenia	3	Early	4	36.98	1.62-843.85	91.7%	RE(HK)
Leukopenia	3	Metastatic	12	18.94	9.49-37.80	26%	RE(HK)
Leukopenia	4	Early	3	NR	NR	NA	NA
Leukopenia	4	Metastatic	7	3.11	2.01-4.82	0%	RE(HK)
Anemia	3	Early	4	4.53	2.82-7.27	0%	RE(HK)
Anemia	3	Metastatic	12	2.73	1.74-4.28	17.4%	RE(HK)
Anemia	4	Early	3	1.0032	0.0628-16.0311	NA†	NA
Anemia	4	Metastatic	3	2.31	0.41-12.98	0%	RE(HK)
Thrombocytopenia	3	Early	4	8.02	3.58-17.95	31.7%	RE(HK)
Thrombocytopenia	3	Metastatic	9	4.49	2.65-7.62	0%	RE(HK)
Thrombocytopenia	4	Early	3	5.38	0.29-99.03	11.8%	RE(HK)
Thrombocytopenia	4	Metastatic	7	1.52	0.69-3.35	0%	RE(HK)

Abbreviations: RR, risk ratio; CI, confidence interval; I^2, heterogeneity; RE(HK), random–effects (Hartung–Knapp); NA, not applicable; NR, not reported.

[a]One study (Hu 2025) contributed two independently randomized cohorts, each analyzed separately and counted as a separate trial.

Table S5. Non-hematological Adverse Events in Early- and Metastatic-Stage Disease

Adverse Event	Grade	Disease Stage	No. of Trials	Pooled effect estimate (RR)	95% CI	I^2(%)	Model
Infections (all types)	3	Early	4	3.00	0.57-15.58	87.2%	RE(HK)
Infections (all types)	3	Metastatic	5	1.82	1.33-2.50	0%	RE(HK)
Infections (all types)	4	Early	3	1.28	0.28-5.75	NA*	FE
Infections (all types)	4	Metastatic	3	4.84	1.60-14.64	0%	RE(HK)
Elevated AST	3	Early	3	4.77	3.28-7.31	49.0%	RE(HK)
Elevated AST	3	Metastatic	10	1.92	1.03-3.61	26.1%	RE (HK)
Elevated AST	4	Early	2	16.55	0.0005-507499.38	0%	RE(HK)
Elevated AST	4	Metastatic	5	1.38	0.27-7.15	0%	RE(HK)
Elevated ALT	3	Early	3	5.37	0.02-1020.38	91.5%	RE(HK)
Elevated ALT	3	Metastatic	10	4.07	2.43-6.81	0%	RE(HK)
Elevated ALT	4	Early	2	5.45	0.02-1311.58	0%	RE(HK)
Elevated ALT	4	Metastatic	6	2.14	0.48-9.43	0%	RE(HK)
Fatigue	3	Early	4	7.76	1.21-49.58	86.9%	RE(HK)
Fatigue	3	Metastatic	8	2.54	1.50-4.32	0%	RE(HK)
Fatigue	4	Early	3	NA	NA	NA	NA
Fatigue	4	Metastatic	13	2.96	0.31-28.42	0%	RE(HK)
Vomiting	3	Early	2	4.02	0.02-789.50	0%	RE(HK)
Vomiting	3	Metastatic	9	1.04	0.51-2.14	13.4%	RE(HK)
Vomiting	4	Early	4	NA	NA	NA	NA
Vomiting	4	Metastatic	13	1.46	0.06-35.34	NA*	FE
Diarrhea	3	Early	4	5.62	0.25-122.72	86%	RE(HK)
Diarrhea	3	Metastatic	7	3.16	0.73-13.61	57.9%	RE (HK)
Diarrhea	4	Early	3	16.91	6.82-41.89	95%	RE(HK)
Diarrhea	4	Metastatic	13	NA	NA	NA	NA
Arthralgia	3	Early	4	0.59	0.27-1.27	52.7%	RE(HK)

Arthralgia	3	Metastatic	7	0.99	0.55-1.78	0%	RE(HK)
Arthralgia	4	Early	3	NA	NA	NA	NA
Arthralgia	4	Metastatic	2	2.12	0.03-148.52	0%	RE(HK)

Abbreviations: RR, risk ratio; CI, confidence interval; I^2, heterogeneity; RE(HK), random–effects (Hartung–Knapp); FE, fixed–effects model; NA, not applicable; NR, not reported.

[a]One study (Hu 2025) contributed two independently randomized cohorts, each analyzed separately and counted as a separate trial.

[b]NA* indicates heterogeneity (I^2) could not be calculated as only single trial reported non-zero event, whereas other trials reported zero events.

[c]NA indicates zero adverse events were reported by the trial.

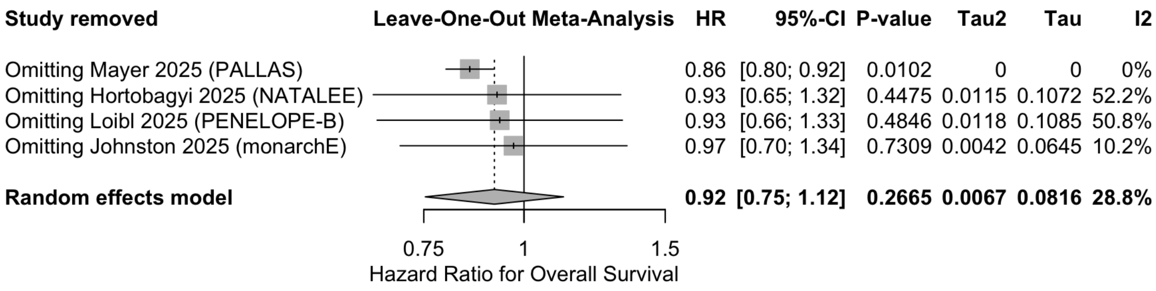

Figure S1. Leave-One-Out Sensitivity Analysis for Overall Survival (OS) in Early-Stage Disease. HR: hazard ratio; CI: confidence interval

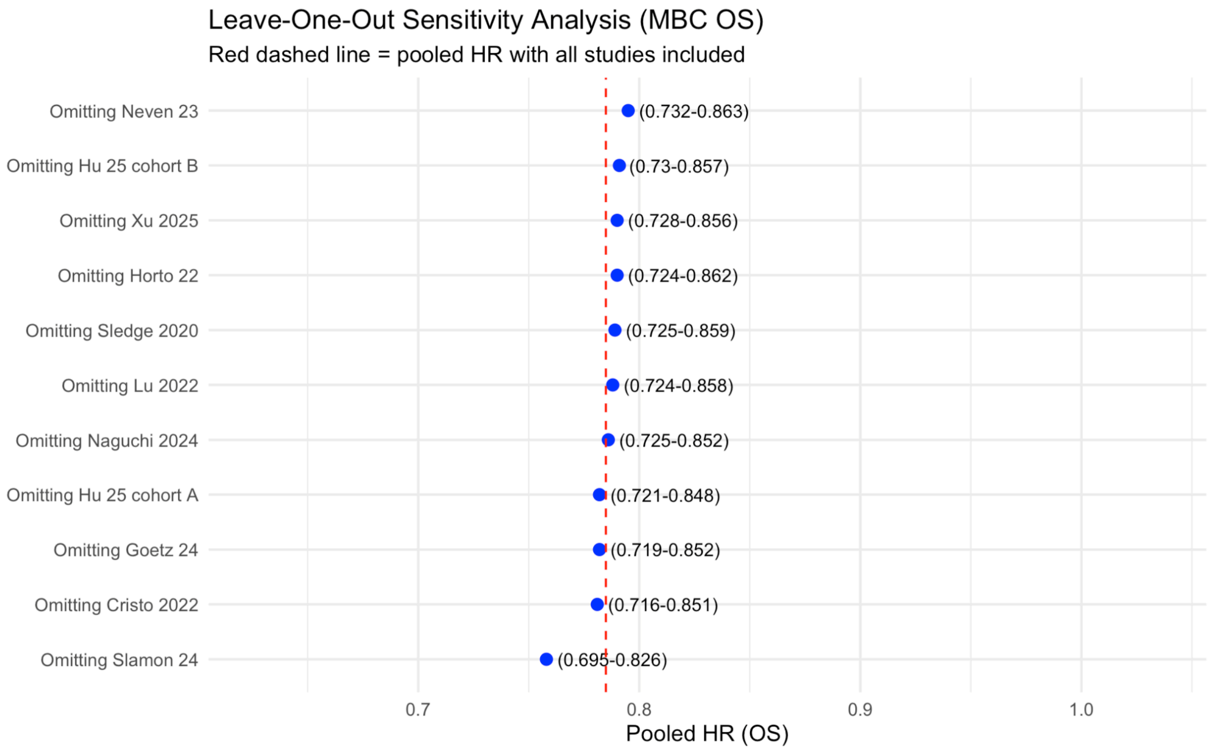

Figure S2. Leave-One-Out Sensitivity Analysis for Overall Survival (OS) in Metastatic Disease. MBC: metastatic breast cancer; OS: overall survival

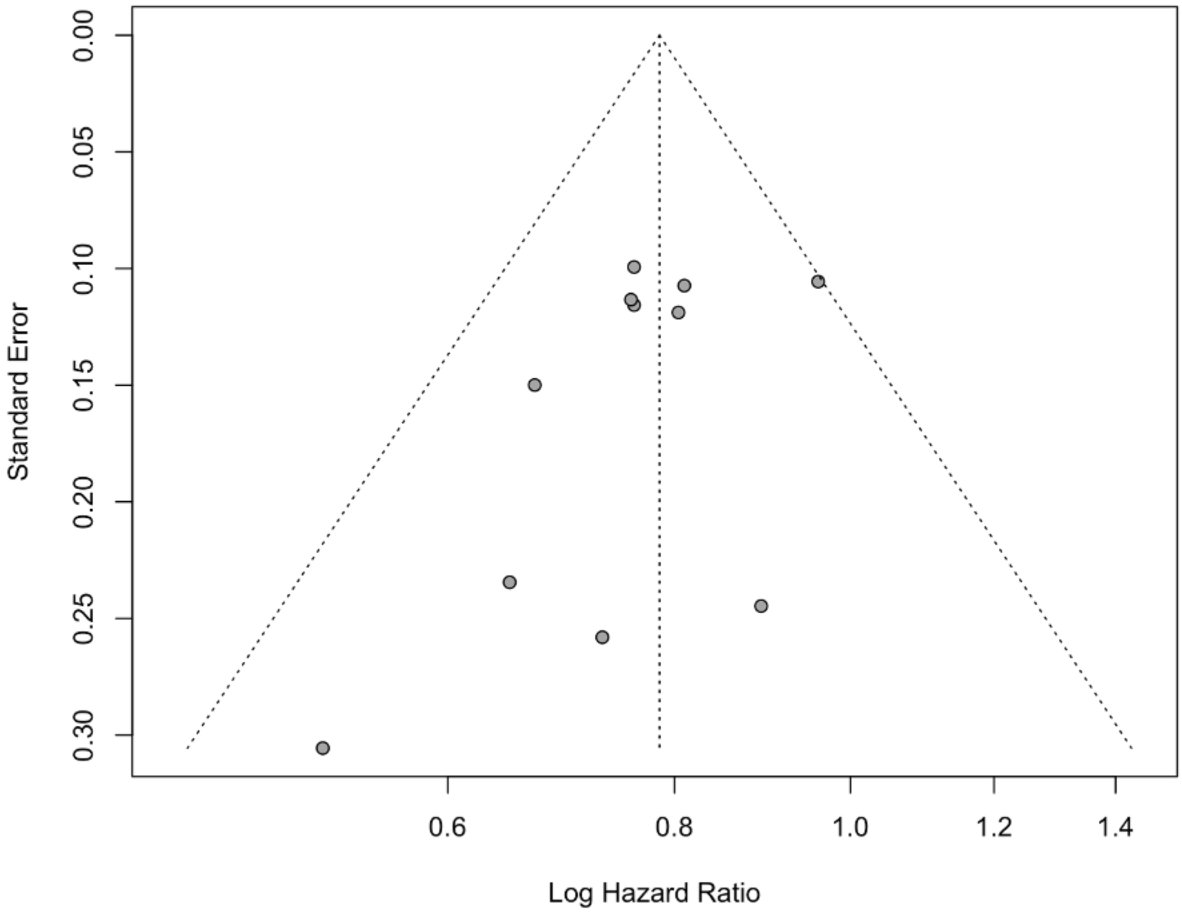

Figure S3. Funnel Plot of Overall Survival (OS) in Metastatic Disease

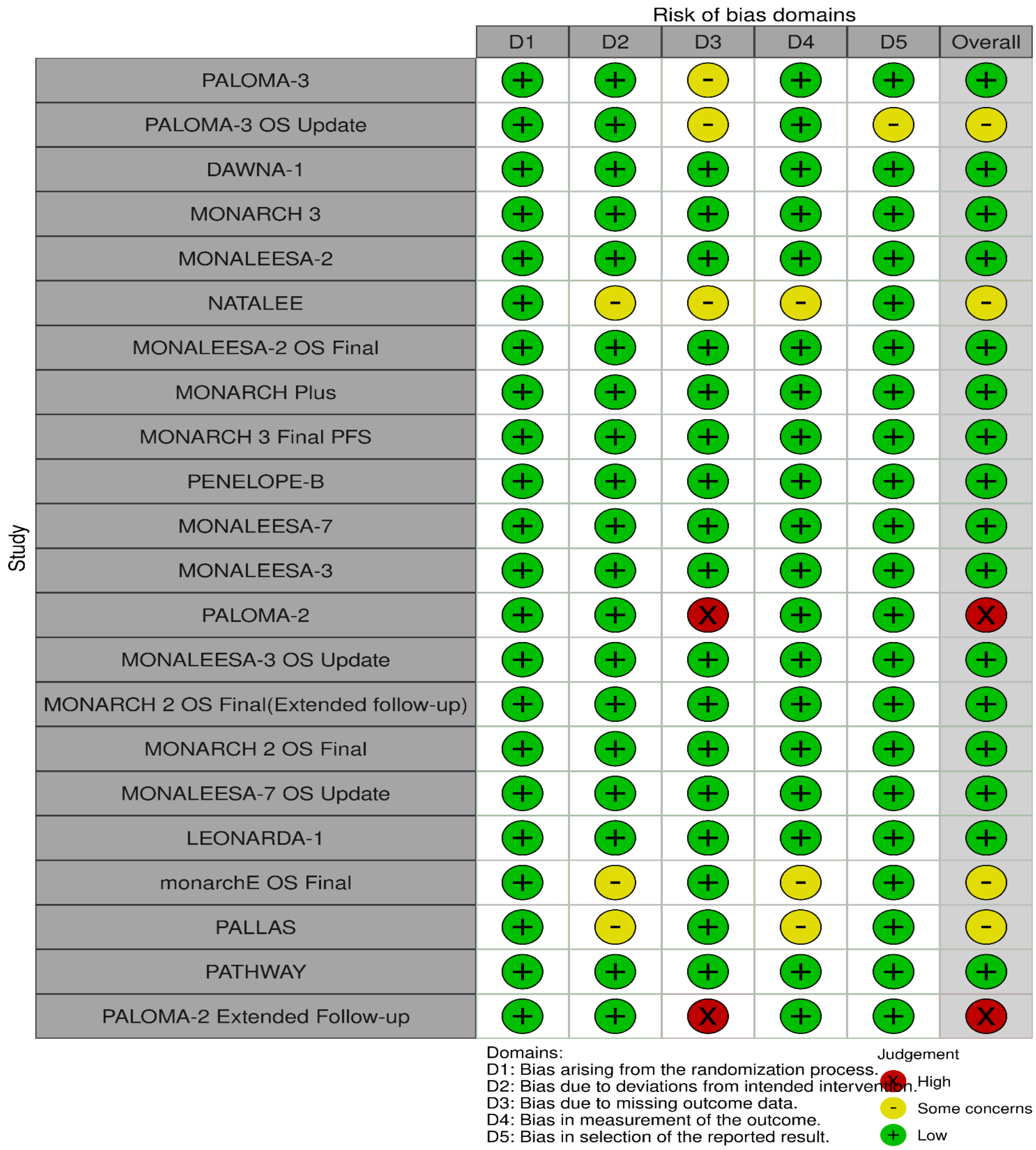

Figure S4. Risk of Bias Assessment in Randomized-Controlled Trials

[a]One study (Hu 2025) contributed two independently randomized cohorts, each analyzed separately and counted as a separate trial.
